# Field trial on a novel control method for the dengue vector, *Aedes aegypti* by the systematic use of Olyset® Net and pyriproxyfen in Southern Vietnam

**DOI:** 10.1186/1756-3305-6-6

**Published:** 2013-01-11

**Authors:** Takashi Tsunoda, Hitoshi Kawada, Trang TT Huynh, Loan Le Luu, San Hoang Le, Huu Ngoc Tran, Huong Thi Que Vu, Hieu Minh Le, Futoshi Hasebe, Ataru Tsuzuki, Masahiro Takagi

**Affiliations:** 1 Institute of Tropical Medicine, Nagasaki University, 1-12-4 Sakamoto, Nagasaki, 852-8523, Japan; 2 Friendship Laboratory (NNFL), National Institute of Hygiene and Epidemiology, No. 1 Yersin Street, Hanoi, Vietnam; 3 Pasteur Institute, 167 Pasteur, District 3, Ho Chi Minh City, Vietnam

**Keywords:** Water container, Dengue vector control, Lid, Long lasting insecticide treated net, Insect growth regulator

## Abstract

**Background:**

Jars, tanks, and drums provide favorable rearing/breeding sites for *Aedes aegypti* in Vietnam. However, the use of insecticides to control mosquitoes at such breeding sites has not been approved in Vietnam since they are also often sources of drinking water, making larval vector control difficult. Mosquito nets pre-treated with long-lasting insecticide treated nets (LLITNs) form an effective measure for malaria control. We examined changes in the abundance of immature *Aedes aegypti* to evaluate the efficacy of covering ceramic jars with lids comprising one type of LLITN, Olyset® Net, in inhibiting oviposition by adult females, and to evaluate the effect of treating other breeding containers, such as flower vases, inside and around the outside of houses with a slow-release pyriproxyfen formulation to kill pupae.

**Methods:**

We selected 313 households for the trial and 363 households for the control in Tan Chanh, Long An province, Vietnam. In the trial area, Olyset® Net lids were used to cover five major types of water container (ceramic jars, cylindrical concrete tanks, other concrete tanks, plastic drums, and plastic buckets), while pyriproxyfen was used to treat flower vases and ant traps. We also monitored dengue virus transmission by measuring anti-dengue IgM and IgG levels in healthy residents in both control and trial areas to estimate the effectiveness of Olyset® Net at controlling the dengue vector, *Aedes aegypti*.

**Results:**

The container-index and house-index for immature *Ae. aegypti* fell steeply one month after treatment in the trial area. Lids with Olyset® Net that fit container openings clearly seemed to reduce the presence of immature *Ae. aegypti* as the density of pupae decreased 1 month after treatment in the trial area. Pyriproxyfen was also effective at killing pupae in the water containers in the trial area. Although the dengue seroconversion rate was not influenced by Olyset® Net, it was lower in two-five year old children when compared to older children and adults in both control and trial areas.

**Conclusions:**

Our study showed that the treatment by Olyset® Net and pyriproxyfen had a strong negative effect on the prevalence of immature *Ae. aegypti*, which persisted for at least 5 months after treatment.

## Background

Dengue is a mosquito-borne disease of increasing public health importance, with over 2.5 billion people at risk globally [1]. More than 100 tropical countries have endemic dengue infections. Dengue hemorrhagic fever (DHF) was first reported in Manila, Philippines, in the early 1950s. It rapidly became an epidemic and spread throughout Southeast Asia within 20 years [2]. Dengue and DHF are primarily urban viral diseases of the tropics, in which the viruses are maintained in a cycle that involves humans and *Aedes aegypti*, a domestic, day-biting mosquito that prefers to feed on humans [3]. As a vaccine for dengue has not yet been developed, controlling its vector in and around the home, where most of the transmission occurs, is the only effective preventive measure currently available. However, spraying insecticides is not an effective way to kill adult mosquitoes unless they are used indoors [4]. The effective way to control *Ae. aegypti* is larval resource reduction, i.e. elimination or cleaning of water containers as habitats for larvae [5].

Dengue fever first appeared in Vietnam in 1959, in Hanoi and Haiphong, and since then has become endemic throughout the country [6]. Poor rural communities in southern Vietnam, like those in other developing countries that do not have access to piped water, have relied mainly on rainwater harvesting systems [7]. In rural areas in Vietnam, water for household use is often stored in 50–2,000 litre containers, most of which are not insect-proof and therefore provide suitable development sites for immature *Ae. aegypti*[8]. Furthermore, even when alternative water supply infrastructure in the form of large tanks, piped water and household taps become available, householders in communities having piped water systems typically still retain their existing water storage containers (mainly 100–200 litre jars), because the piped water supply is not always reliable [8]. Ceramic jars, tanks, and drums provide suitable rearing/breeding sites for *Ae. aegypti* in Vietnam [6,9,10]. Treatment of such breeding sites with insecticides based on organophosphates or insect growth regulators, some of which are recommended for the treatment of drinking water by WHO, seems to be the best and most convenient measure. However, the use of insecticides to control mosquitoes at their breeding sites has not been approved for the treatment of drinking water in Vietnam, which uses community-based biological control strategies for this purpose, for example, by the use of the copepod, *Mesocyclops*[6,11,12]. However, this form of control is lost from containers when all the water is used up or the containers are cleaned [13], which then makes larval vector control difficult. Therefore, alternative measures are necessary, especially when mosquito control by *Mesocyclops* fails.

While it is important to render rearing habitats unusable for immature stages in the control of dengue vector mosquitoes, it is perhaps even more important to prevent gravid females from entering breeding habitats (e.g. containers) in the first place. There have been a few studies in which vector populations have been successfully suppressed by covering the breeding containers [14], thereby creating a physical barrier that prevents female mosquitoes from entering the containers [15]. In addition, mosquito nets treated with synthetic pyrethroids have proved to be a successful intervention tool for malaria [16-19] and other vector-borne diseases [20] such as dengue [14,21].

Olyset® Net was developed as a bed-net to protect people from being bitten by malaria vectors [22,23]. For example, surveys in India showed a 45.7% reduction in malaria prevalence in villages using Olyset® Net [23]. Olyset® Net was also effective for the control of *Ae. aegypti*[24]. This long-lasting insecticidal net can often remain effective for over 5 years in the home [17].

The purpose of our study was to determine whether the study of covering the lids of domestic water storage containers with Olyset® Net would help in controlling *Ae. aegypti* in southern Vietnam where over 80% of dengue cases of the country are reported each year, and where *Ae. aegypti* may be more abundant than in the northern and central parts of Vietnam [11]. Flower vases and ant traps are also important breeding sites for *Ae. aegypti*[7,25]. In addition, we tested the effectiveness of applying an insect growth regulator to flower vases and ant traps inside and around the outside of houses. Finally, we examined the abundance of immature *Ae. aegypti* before and after intervention, and also compared the change in abundance between the trial and control areas.

## Methods

### Study site

Tan Chanh, a commune of Can Duoc district in Long An province, is located 30 km south of Ho Chi Minh City, Vietnam. It has two distinct seasons. The rainy season starts in May and ends in November, and the dry season begins in December and lasts until April. Tan Chanh’s seven hamlets contain 300 to 400 houses each and a population of 1,400 to 2,000, giving the commune a total of around 12,000 residents in 2,540 houses. There were 30 dengue patients in 2007 and 104 in 2008 from this commune [26,27].

Tan Chanh is located along the Vam Co river and near a seaport. The water in the rivers and canals in the commune is saline from December to June and is fresh from July to November. The Government of Vietnam has committed to providing rural communities with increased access to safe water through a variety of household water supply schemes including wells, water tanks and ceramic jars, and tap water schemes [28]. The tap water is actually supplied by a private company, and is not sufficient for all the residents for their daily use and furthermore, is sometimes stopped. The locals also typically cannot afford to dig their own wells. Therefore, they become resourceful and collect rain-water and store it in 100–200 litre ceramic jars and big tanks.

### Insecticide

Insecticides were presented in two media, the first being a controlled-release, plastic net impregnated with 2% permethrin (Olyset® Net). The second medium was the EcoBio-Block® S, a novel controlled-release system for the insect growth regulator, pyriproxyfen. Each block is composed of a porous volcanic rock and cement into which the aerobic groups of *Bacillus subtilis natto* are incorporated as a water purifying agent [29]. Specifically, each EcoBio-Block® S was produced by mixing neutralized cement (40.6% w/w), aggregate (pumice stone, 52.1% w/w), a mixture of aerobic bacteria and nutrient medium (4.1% w/w), and 0.5% granular formulation of pyriproxyfen (Sumilarv® 0.5 G, Shinto Fine Co., Ltd., Osaka, Japan, 3.2% w/w). The content of pyriproxyfen in the block was 0.016% (w/w).

### Study design

**Figure 1:**
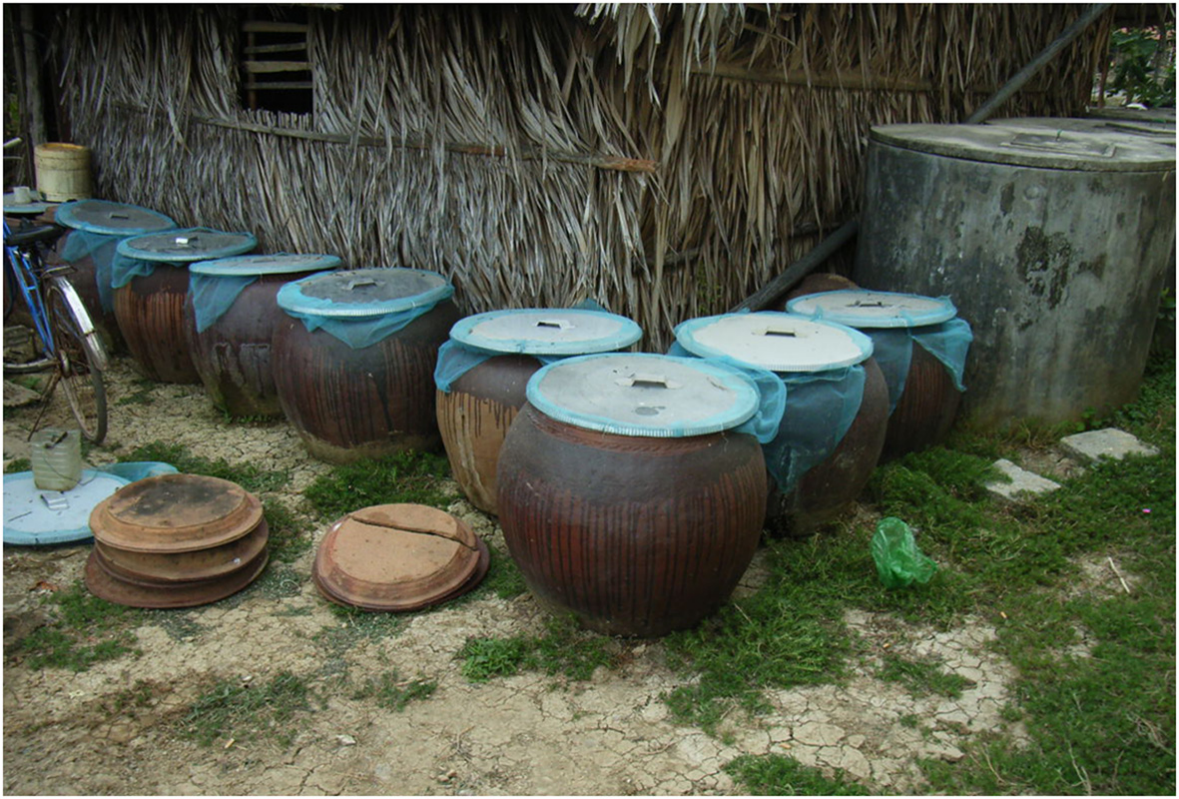
Metal water jar lids covered with Olyset^®^ Net.

We selected one hamlet (313 households in total) as a trial area and two hamlets (363 households in total) as a control area (which received no interventions) in Tan Chanh commune. In the trial area, five major water containers (ceramic jars, cylindrical concrete tank, other concrete tanks except for those set in toilets, plastic drums, and plastic buckets) were covered with lids fitted with Olyset® Net in early September, 2008 (Figure 1). A 30 × 150-cm piece of net was tied around the circumference of the lid. Additionally, ant traps and flower vases inside and around the outside of houses were treated with EcoBio-Block® S pyriproxyfen by crushing a block into small pieces (less than 1 cubic cm) and putting them in the container at the rate of approximately 1 g per 1 liter of water.

### Entomologic survey

Mosquito populations were assessed before treatment, and one, three and five months after treatment in the selected houses of control and trial areas. All the houses were numbered in the control and trial areas, and 71 houses were randomly selected in August and 50 houses in October, December, and February. The assessment entailed collecting immature mosquitoes inside and outside houses as described in Tsuzuki et al. [10]. Briefly, ceramic jars, concrete basins, and toilet concrete basins were sampled by sweeping through them five times with a small net [30], and small containers were upturned to collect pupae. All water-holding containers both indoors and outdoors, were inspected only after obtaining the necessary permission from the occupants. The house index, container index, and the number of pupae from the trial and control houses were compared to determine the efficacy of the insecticides. Containers with one or more *Ae. aegypti* immatures were defined as immature positive containers. The lids (covers) were examined from all the water storage containers in the selected households and were categorized into three types; no lid (NL), a lid with a gap or an opening through which the water surface in the containers was visible (GL), a lid that fitted an opening (FL). A sample of the pupae collected was examined in the laboratory and allowed to develop into adult mosquitoes, which were then identified by species and sex.

### Monitoring of dengue virus transmission

Dengue virus transmission was monitored at both control and trial areas. Households with more than five family members were selected and 2 ml blood samples collected twice from each healthy family member between the age of 2 and 65 years. Preliminary observations showed that only large families (with more than five members) used jars or concrete tanks to store water. From the trial area, 301 and 222 blood samples were collected in September 2008 and February 2009, respectively. A total of 352 and 286 blood samples were collected at the control area in September 2008 and February 2009, respectively. Since there was a delay in the processing of the application to collect blood samples at the Ministry of Health in Vietnam, the first blood collection was one month after the beginning of the trial. Written informed consent was obtained from all participants. All serum specimens were subjected to in-house IgG and IgM capture enzyme linked immunosorbent assay (ELISA) [31,32] and indirect IgG-ELISA at 1:100 and 1:400 dilution ratios, respectively, using tetravalent dengue virus antigen for determination of seroconversion rates in populations from both control and trial areas. Seroconversion was determined by positive conversion of the second blood sample after IgM ELISA or a four-fold elevation of IgG titre of the second blood sample, when compared to the first blood sample. All possible dengue cases were reported to the Virology Department, Pasteur Institute Ho Chi Minh City from August 2008 to February 2009.

### Ethics

The study protocol was reviewed and approved by both the Scientific and Ethics Committees of the Pasteur Institute, Ho Chi Minh City, and the Ethics Committee of the Institute of Tropical Medicine, Nagasaki University. Written informed consent was obtained from all the participants for the collection of the samples and their subsequent analysis. Participation by children required consent from at least one parent and the child’s assent. The children and their families from whom blood was taken were insured for six months.

### Statistical analysis

We examined the association between the response variable, abundance (presence/absence) of immature *Ae. aegypti* and explanatory variables, area (control/trial) and state of lid (NL, GL, FL; see Entomological Survey above). Multivariate logistic regression modeling was used to calculate odds ratios and 95% confidence intervals (95% CIs). We also used multivariate logistic regression modeling to examine the serology conversion. Explanatory variables were area (control/trial), age (two to five years old/over five years) and gender (male/female). We constructed the full model including all combinations of interactions and sequentially eliminated an interaction and/or variable, based on the Akaike Information criterion (AIC) value. When the best model included an interaction, analyses were stratified by area or gender. Multicollinearity was assessed by using the variance inflation factor and a variance inflation factor greater than 5.0 was considered to be a possible cause for concern [33].

The number of pupae per container was log_e_ transformed to the normal after adding 0.5 to all the values, prior to statistical analysis [34]. The number of pupae per container within each month was tested using ANOVA where area and the state of lid were included as effects.

The G-test for independence was used to compare the frequency of lid type between trial and control areas and to test the effect of pyriproxyfen on the number of pupae. All statistical analyses were performed using the R statistical software 2.6.2 [35].

## Results

### Comparison of entomological indices between trial and control areas

We examined 3,869 containers in the trial area and 4,198 in the control area from August 2008 to February 2009 (Table 1). Ceramic jars were the most common containers (78.9-82.4% in the trial area and 81.3-98.4% in the control), followed by plastic drums and ant traps in both areas. Five major types of water container (ceramic jar, cylindrical concrete tank, other concrete tank, plastic drum, and plastic bucket) made up 92.0% (n=811) of all containers in the trial area in October, 94.7% (n=874) in December, and 92.1% (n=727) in February. The relative percentages of clay bowls, gardening pots, concrete toilet tanks, and discarded containers were low in both areas.

**Table 1:** Relative percentages of various container types observed in Tan Chanh commune from August 2008 to February 2009

**Container**	**Trial**				**Control**			
	**Aug**	**Oct**	**Dec**	**Feb**	**Aug**	**Oct**	**Dec**	**Feb**
Ceramic jar	82.2	82.4	81.0	79.8	83.8	81.7	98.4	81.3
CCT^1)^	3.4	4.0	3.1	4.6	0.6	1.2	1.0	1.2
OCT^2)^	0.2	1.0	1.8	0.8	0.6	0	0.3	0.1
PD^3)^	6.5	2.8	4.2	3.5	5.3	10.7	4.7	9.0
PB^4)^	0.3	1.7	4.4	3.4	2.6	1.0	1.5	3.0
Vase	1.3	1.5	1.0	2.8	0.8	0.8	1.0	1.4
Clay bowl	0.5	0.1	0.2	0.3	0.3	0.2	0	0
Plant pot	0	0	0	0.4	0.1	0.2	0.8	0.4
TCT^5)^	0.4	0	0	0.1	0	0	0	0
Ant trap	4.0	5.9	2.6	4.1	2.9	3.5	1.9	2.8
Discarded	0.8	0.5	1.2	0	2.8	0.8	1.4	0.3
Others	0.4	0.1	0.3	0.3	0.1	0.1	0	0.5
Total (%)	100	100	100	100	100	100	100	100
Total (No.)	1275	882	923	789	1306	1031	927	934

^1)^ CCT; Cylindrical concrete tank.

^2)^ OCT; Other concrete tank.

^3)^ PD; Plastic drum.

^4)^ PB; Plastic bucket.

^5)^ TCT; Toilet concrete tank.

**Figure 2:**
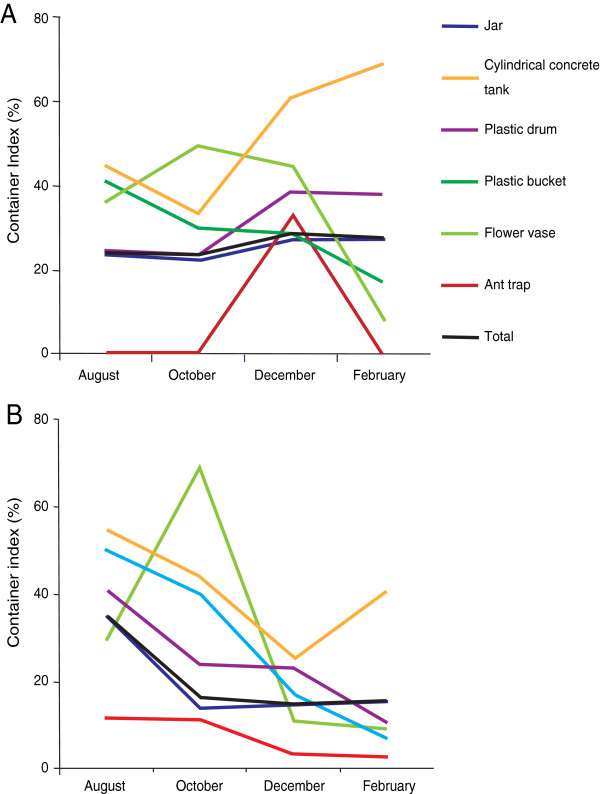
**Container index of various containers for the period August 2008 to February 2009.** (**A**) Trial area. (**B**) Control area. The treatment was carried out in September, 2008.

The Container Index in the trial area gradually decreased from October to February (Figure 2B), when compared to the CI in the control area (Figure 2A), except for the cylindrical concrete tanks in February. The total container index in the trial area decreased in October and continued to be under 20% until February.

**Figure 3:**
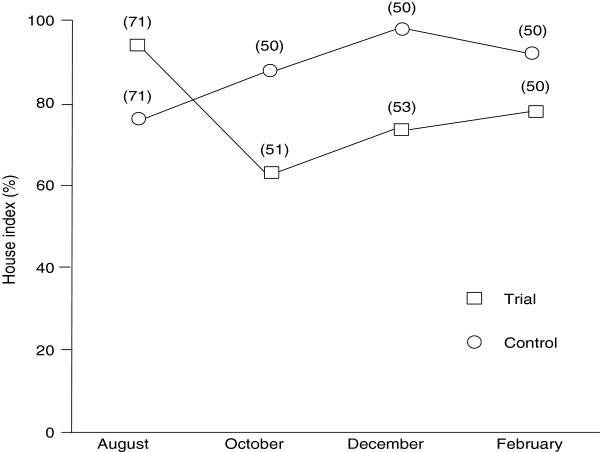
**House index in trial (squares) and control (circles) areas from August 2008 to February 2009.** The numbers of houses examined are given in parentheses. The treatment was carried out in September, 2008.

The house index (HI) of the trial area was higher than that of the control area in August, in general (Figure 3). The HI of the trial area suddenly decreased in October and gradually increased from December to February, whereas the HI of the control area increased gradually from August to December.

### Effect of lid and insecticide treatment

The proportion of containers with fitted lids in the trial area (36.6%) was lower than that in the control area (49.6%) in August. However, the proportion of fitted lids was higher in the trial area (78.5-81.3%) than in the control area (38.9-55.5%), from October to February. Most of residents used lids with Olyset® Net properly in the trial area, although some did not use lids at all or used them inappropriately despite of instructions.

The numbers of positive containers with *Aedes* immature stages were higher in the trial area than in control area in August (Table 2). However, they were lower in the trial area when FL and even GL lids were used, when compared to the control area. The final model was different between August and October on the one hand, and December and February on the other, because of the significant interaction between the area and state of lid in December and February (Table 3a, b). In August and October, the main effects of area and lid were important determinants of the presence of *Aedes* immature stages. Although the odds ratio of the trial area was 70% higher than that of the control area before treatment (August), it reduced to 70% after treatment (October). The odds ratio of the GL lids was 30% lower than that of the FL lids in August, although it was 30% higher than that of the FL lids in October. Likewise, in comparison with FL, the odds ratio of no lids (NL) increased from 1.3 in August to 2.7 in October. As the model to best explain the data for December and February incorporated the interaction of the area and state of lid, the odds ratio was calculated separately by area. In both areas, odds ratios of containers with GL lids and NL containers were at least 80% higher than those of containers with FL lids. The odds ratio of NL containers was 5.0 in the trial area in December and 5.6 in the control area in February.

**Table 2:** **The percentages of positive containers (for the presence of *Aedes *immature stages) listed by lid type for each month in the trial and control areas from August to February**

		**Trial**		**Control**	
**Month**	**Lid type**^**1)**^	**No. of containers**	**Positive containers (%)**	**No. of containers**	**Positive containers (%)**
August	NL	332	40.7	308	29.5
	GL	417	28.5	304	16.8
	FL	432	34.3	602	23.9
October	NL	85	32.9	203	34.0
	GL	67	13.4	231	22.1
	FL	659	12.0	541	17.9
December	NL	84	38.1	221	33.5
	GL	94	20.2	318	33.3
	FL	696	10.9	341	20.2
February	NL	90	37.8	239	35.6
	GL	66	19.7	201	34.8
	FL	571	9.8	363	22.6

^1)^ NL; no cover, GL; gapped lid, FL; a fitted lid.

**Table 3:** **The relative risk, as given by the odds-ratio, for the lid types and areas for the presence of *Aedes *immature stages in (a) August and October and (b) December and February**

**(a) August and October**				
**Month**	**Risk factor**		**Odds ratio (95% CI)**	***P***
August	Area			
	Control		1.0	
	Trial		1.7 (1.4-2.1)	<0.001
	Lid^1)^			
	FL		1.0	
	GL		0.7 (0.6-0.9)	<0.01
	NL		1.3 (1.1-1.6)	<0.05
October	Area			
	Control		1.0	
	Trial		0.7 (0.5-0.9)	<0.01
	Lid^1)^			
	FL		1.0	
	GL		1.3 (0.9-1.8)	0.1
	NL		2.7 (2.0-3.6)	<0.001
**(b) December and February**				
**Month**	**Site**	**Risk factor**	**Odds ratio (95% CI)**	***P***
December^1)^	Control	Lid^3)^		
		FL	1.0	
		GL	2.0 (1.4-2.8)	<0.001
		NL	2.0 (1.4-2.9	<0.001
	Trial	Lid^3)^		
		FL	1.0	
		GL	2.1 (1.2-3.6)	<0.05
		NL	5.0 (3.0-8.3)	<0.001
February^2)^	Control	Lid^3)^		
		FL	1.0	
		GL	2.3 (1.2-4.4)	<0.01
		NL	5.6 (3.4-9.3)	<0.001
	Trial	Lid^3)^		
		FL	1.0	
		GL	1.8 (1.3-2.7)	<0.05
		NL	1.9 (1.3-2.7)	<0.001

^1)^*P* value for interaction = 0.01.

^2)^*P* value for interaction = 0.003.

^3)^ NL; no cover, GL; a lid with a gap, FL; a fitted lid.

^1)^ NL; no cover, GL; a lid with a gap, FL; a fitted lid.

The number of containers not treated with Olyset® Net ranged from three to 36 (Table 4). In the trial area, containers with Olyset® Net had a significantly lower percentage of *Aedes* immature stages positive than those with no Olyset® Net in October. The probability of the presence of *Aedes* immature stages was also significantly different between containers with Olyset® Net and those without Olyset® Net, when December and February were considered together.

**Table 4:** **Effect of Olyset® Net on the presence of *Aedes *immature stages in (a) October, (b) December and February**

**(a) October**				
**Olyset® Net**	**No. of positive containers**	**No. of negative containers**	***χ***^**2**^	***P***
Use	73	617	31.04	<0.001
No use	15	21		
**(b) December and February**				
**Olyset® Net**	**No. of positive containers**	**No. of negative containers**	***χ***^**2**^	***P***
Use	156	1240	6.38	<0.05
No use	8	23		

The G test for independence was performed between containers with Olyset® Net and those with no Olyset® Net. As the number of containers with no Olyset® Net was under 5 in December, the data from December was added to February.

**Figure 4:**
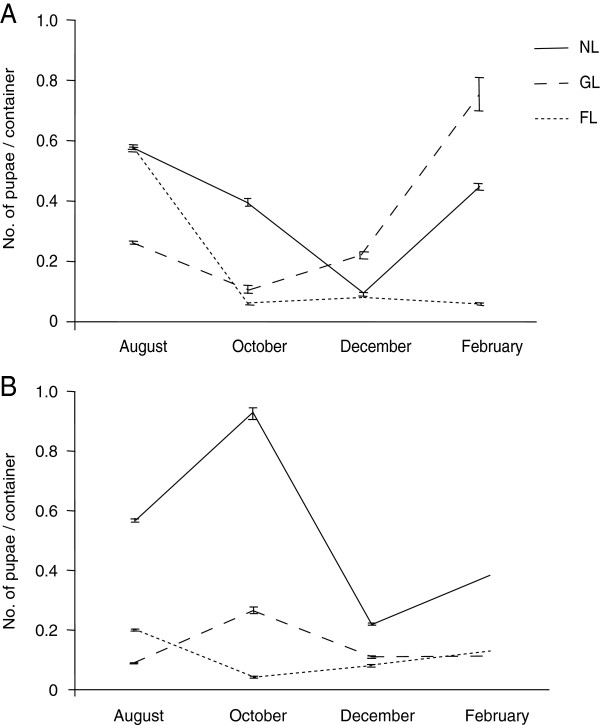
**A comparison of the density (mean±S.E.) of pupae among different types of containers: those with no lid (NL), with gapped lids (GL), and with fitted lids (FL) from August 2008 to February 2009.** (**A**) Trial area. (**B**) Control area. The treatment was carried out in September, 2008.

The number of pupae per container decreased for all three states of lid in the trial area in October (Figure 4A). On the other hand, the density of pupae in containers with GL lids as well as the NL containers increased in October in the control area (Figure 4B). ANOVA revealed a significant effect of the type of lid on the number of pupae per container, each month. The main effect of area was significant only in August (*P*<0.01). The main effect of lid type was significant in all months at least at the 5% level. The two-way interactions were significant only in February (*P*<0.05). The density of pupae collected from the fit lid was under 0.1 per container in the trial area and under 0.2 per container in the control area from October to February. However, in the trial area, the density of the pupae in the containers with GL lids increased in December and reached to 0.76 per container in February. The ANOVA results also showed that interaction between area and lid was significant only in February.

### Effect of IGR

More pupae were dead in the trial area (n=81) than in the control area (n=9) after treatment. There was no difference between the trial and control areas in the effect of pyriproxyfen on the number of live *Aedes* pupae before treatment. There were no dead pupae in containers without pyriproxyfen in the trial area, since some resident forgot to keep pyriproxyfen in flower vases and ant traps. The containers treated with pyriproxyfen saw significant reduction in the number of pupae that would emerge into adults when compared to those with no treatment (χ^2^test; *P*<0.001).

### Dengue virus transmission

Seroprevalence rates of anti-dengue IgM and IgG in the healthy residents were not significantly different between the trial and control areas. Dengue seroconversion was observed in 23 (62.2%) of 37 seronegative cases in the trial area and in 44 of 59 (74.6%) seronegative cases in the control area. A model with age as the only explanatory variable was the best one (*P*=0.03), which means that neither area (control/trial) nor gender (male/female) showed an effect on dengue seroconversion in our study. The dengue seroconversion rate in children 2–5 years old was lower than in those over 5 years old (Table 5). The odds ratio of these was 0.25 (95% CI: 0.07-0.88), indicating that the risk of dengue in 2–5 year olds was a quarter of that for those over 5 years.

**Table 5:** Dengue seroconversion rate at the trial and control areas

**Area**	**Sex**	**Age (years)**	**No. at the 1st **^**1)**^	**No. at the 2nd **^**2)**^	**Seroconversion rate (%)**
Trial	Male	2-5	1	0	0
		>5	15	12	80.0
	Female	2-5	6	2	33.3
		>5	15	9	60.0
Control	Male	2-5	2	1	50.0
		>5	34	25	73.5
	Female	2-5	3	2	66.6
		>5	20	16	80.0

^1)^ Number of samples negative for dengue IgG at the 1^st^ blood collection.

^2)^ Number of samples with seroconversion (i.e., positive for dengue IgG) at the 2^nd^ blood collection.

## Discussion

Our study showed that Olyset® Net and pyriproxyfen was successful in the control of *Ae. aegypti* immature stages, although seroprevalence rates were not significantly different between the trial and control areas. While more containers had immature mosquitoes in the trial area than in the control area before treatment, the number of positive containers and the number of pupae in the trial area were both less than in the control area after Olyset® Net and pyriproxyfen treatment, which suggests that the suppression of mosquitoes can be attributed to the treatments tested.

The container types favorable for *Ae. aegypti* immature stages can be dramatically different among local areas [36-38]. The ceramic jar was the main container to store rain water and the most important breeding container for *Ae. aegypti* in Tan Chanh. Compared to other provinces, the prevalence, i.e. the proportion among all the containers, and the container index of ceramic jars is higher in southern Vietnam [9,25]. In our study, the prevalence of ceramic jars was more than 80% and its container index was 35% before intervention in the trial area of Tan Chanh. However, the prevalence and container index of ceramic jars were 30.2% and 26.0%, respectively, in Hanoi City (northern Vietnam) [9] and 5.9% and 16.3% in Nha Trang City (central Vietnam) [9,25]. Thus, it is important to suppress immature stages mainly in ceramic jars as the dengue control strategy in southern Vietnam.

People in southern Vietnam store water because of the unreliable water supply and/or distaste for tap water [8]. In many areas throughout Vietnam the contribution of poorly designed or maintained water tanks to *Ae. aegypti* proliferation is well recognized [39]. However, as it is difficult to drain the heavy containers completely, a small amount of water invariably remained at the bottom of some ceramic jars in Tan Chanh, underscoring a practical difficulty, even though the jar is recognized as a breeding habitat of vector mosquitoes by the locals.

Unfortunately, most of villagers prefer not to cover their water containers, and furthermore, the government supplies insufficient number of lids for outdoor containers [37]. Our results indicate that containers with FL lids, which ensured a complete cover over the opening, was effective in reducing the positive rate of containers with *Aedes* immature stages. However, more covered ceramic jars were infected with *Ae. aegypti* immature stages than uncovered ones located outdoors [15]. Gravid *Ae. aegypti* are known to find small gaps between the lid and the lip of the ceramic jars, as seen with those that were covered by an unmodified commercial aluminum lid in four of six trials in laboratory cages [40]. In our study, the density of pupae in containers with GL lids in the trial area was the highest in February. These results suggest that GL lids attract gravid females, which then lay eggs inside the container, as GL lids make more shade than NL lids.

In a laboratory test, Olyset® Net reduced landing attempts of mosquitoes and increased the flying time [41], which suggests that the Olyset® Net operates as a repellent. Our results showed that Olyset® Net reduced the number of positive containers with *Aedes* immature stages, which suggests that females avoided laying eggs in containers with Olyset® Net.

The percentage of properly used insecticide-treated (IT) covers was high for 5 months in the trial area in our study. A study in Cambodia shows that when about 88% of IT cover was used properly, a 58% reduction in the pupa per person index was shown at 13 weeks after treatment [21]. Researchers did not find a significant effect of IT covers on entomological indices in north-west Venezuela because the usage of IT cover was low [42]. In light of this study, our findings suggest that a widespread and proper use of IT covers is critical for the suppression of *Ae. aegypti*.

In addition to IT covers, we used pyriproxyfen, insect growth regulator (IGR), in flower vases and ant traps in the trial area. In Vietnam, people offer flowers on an altar on the first and fifteenth days of the lunar calendar. It takes 13 days for *Ae. aegypti* to develop in a temperature range of 20-27°C [43]. As the average lowest temperature is 22°C in Ho Chi Minh City [44], adult mosquitoes may emerge before the next floral tribute. Therefore, it is necessary to add IGR to the flower vase when people change water, or the water needs to be cleaned or exchanged at least once in the 15 day interval.

Dengue infection rates among young children and women have sometimes been higher than in older children and adult males [45,46]. Our monitoring of anti-dengue IgM and IgG in healthy residents in both control and trial area revealed seroconverted cases in both the trial and control area. A lower seroconversion rate in the trial area was evident in children 2–5 years old but there were no clear differences between the men and women of our study, which indicates that our treatment was effective on young children since they stay home most of the time. The seroconversion rates at the trial area were lower than the rates observed in the control area but not enough to be statistically significant. Further research is needed to clarify exactly how human age and gender are related to the seroconversion rate.

## Conclusions

In summary, we fixed Olyset® Net to the lids of water containers to reduce the density of immature *Ae. aegypti*, since the use of insecticides to control mosquitoes at their breeding sites has not been approved for the treatment of drinking water in Vietnam. Pyriproxyfen, an insect growth regulator, was also applied to small water containers such as flower vases to kill mosquito pupae. Our study showed that use of Olyset® Net and pyriproxyfen provided effective control of *Ae. aegypti* and an intact lid also contributed to the reduction of the density of *Ae. aegypti*, which can lead to the suppression of dengue epidemics.

## Competing interests

The authors declare that they have no competing interests.

## Authors’ contributions

HK, TH, LLL, SLH, HNT, HTQV, FH, AT, and MT participated in the study design and coordination. TT, HK, TH, LLL, HTQV, HML, FH, and AT carried out the experimental work. TT, HK, and TH conducted the statistical analysis. TT, HK, and FH drafted the manuscript. All authors read and approved the final manuscript.
